# Influence of postpartum onset on the course of mood disorders

**DOI:** 10.1186/1471-244X-6-4

**Published:** 2006-01-26

**Authors:** Alessandro Serretti, Paolo Olgiati, Cristina Colombo

**Affiliations:** 1 Department of Psychiatry, Vita-Salute University, San Raffaele Institute, Milan, Italy

## Abstract

**Background:**

To ascertain the impact of postpartum onset (PPO) on the subsequent time course of mood disorders.

**Methods:**

This retrospective study compared per year rates of excited (manic or mixed) and depressive episodes between fifty-five women with bipolar (N = 22) or major depressive (N = 33) disorders with first episode occurring postpartum (within four weeks after childbirth according to DSM-IV definition) and 218 non-postpartum onset (NPPO) controls. Such patients had a traceable illness course consisting of one or more episodes alternating with complete symptom remission and no additional diagnoses of axis I disorders, mental retardation or brain organic diseases. A number of variables reported to influence the course of mood disorders were controlled for as possible confounding factors

**Results:**

Bipolar women with postpartum onset disorder had fewer excited episodes (p = 0.005) and fewer episodes of both polarities (p = 0.005) compared to non-postpartum onset subjects. No differences emerged in the rates of depressive episodes. All patients who met criteria for rapid cycling bipolar disorder (7 out of 123) were in the NPPO group. Among major depressives, PPO patients experienced fewer episodes (p = 0.016). With respect to clinical and treatment features, PPO-MDD subjects had less personality disorder comorbidity (p = 0.023) and were less likely to be on maintenance treatment compared to NPPO comparison subjects (p = 0.002)

**Conclusion:**

Such preliminary findings suggest that PPO mood disorders may be characterized by a less recurrent time course. Future research in this field should elucidate the role of comorbid personality disorders and treatment. Moreover it should clarify whether PPO disorders are also associated with a more positive outcome in terms of social functioning and quality of life.

## Background

The lifetime time course of mood disorders ranges from a single episode of illness to a recurrent pattern with few or no intervals [1]. This heterogeneity would suggest abundance of course predictors. To date, however, despite considerable efforts by several research groups, only a few of them have been identified. The strongest prognostic factor is the number of previous affective episodes [2,3]. There is mounting evidence that cycle length gets progressively shorter within the first three to five episodes, then it approaches a level corresponding to the maximum individual frequency of episodes [3]. Therefore episode number is a late predictor. On the contrary it would be desirable to influence illness process before it comes to its peak. This necessarily leads to search early predictors, most of which should be related to the first episode.

Amongst such variables polarity has probably been investigated the most: a number of studies have consistently demonstrated that bipolar disorders with manic or mixed onset are associated with a worse outcome than depressive onset forms, although such a difference seems to disappear with illness duration [4-6].

The role of age at onset is more controversial: some authors have indicated a worse prognosis for early onset patients [7] but others failed to confirm this finding [8] or even reported an association in the opposite direction [9]

The presence of psychotic symptoms has also been associated with a higher rate of relapse and a rapid deteriorating course [10-13], along with other descriptors such as family history of psychiatric illness [14], comorbidity with anxiety or personality disorders [15] and substance abuse [16].

The aim of this study was to assess the influence of postpartum onset on mood disorder course.

## Methods

### Subjects

This retrospective study is based on the data of a large population of women consecutively admitted to the Center for Mood Disorders of S. Raffaele Hospital (HSR-CMD) in Milan during the period between January 1990 and January 2000. These subjects have participated in previous genetic and clinical trials undertaken by our research group [17-21] and we remand to those publications for a thorough description of their recruitment. Informed consent was obtained after the procedure was explained to subjects and studies were performed after approval of the local ethical committee.

Eligible subjects were bipolar I and major depressive women aged between 15 and 44 years at illness onset who had a traceable illness course characterized by one or more affective episodes alternating with complete remission defined as a HDRS_17 _score <7 [22] and no symptoms of DSM-IV hypomanic, manic or mixed episodes [23]. Their minimum length of illness was 1 year calculated from the onset of the intake episode to study entry. Rapid cycling, the occurrence of ≥ 4 episodes within one year [24], was not considered among exclusion criteria. Instead additional diagnoses of mental retardation, substance-related disorders or other Axis I disorders and the presence of any organic disease impairing psychiatric evaluation were considered exclusion criteria. Moreover we excluded all patients without reliable treatment histories.

1,080 women charts were reviewed to be included in the study, of whom 125 had their first episode during the postpartum period (within 4 weeks after childbirth according to DSM IV definition) [23]. By applying the selection criteria mentioned above the study sample was reduced to 55 women with postpartum onset (PPO) mood disorders and 218 non-postpartum onset (NPPO) controls. These subjects did not significantly differ from the original population as for age, diagnostic subtype (BP vs MDD) and familiarity.

### Diagnostic assessment and data collection

Lifetime diagnoses were assigned by two independent psychiatrists according to DSM-III-R and DSM-IV criteria [23,25] on the basis of structural clinical interviews – SCID version 1.0 [26], SCID-I/P version 2.0 [27] and SCID II for personality disorders [28] – and complementary information gathered from the patients' relatives, other health professionals and, when available, medical records [29]. The treating psychiatrist proposed preliminary diagnoses of Axis I and Axis II disorders which had to be confirmed by a senior psychiatrist blind to his colleague's evaluation. If there was no agreement, a second senior psychiatrist was involved. If disagreement persisted, the case was ruled out. Actually no patient was excluded because of discordant classification.

A similar consensus procedure was employed to ascertain illness time course and treatment history. Through a method similar to the LIFE technique [30], except for a longer follow up period, the two psychiatrists produced detailed life-charts accounting for the number and characteristics of all affective episodes. These included age of onset, DSM-IV type, severity (1 to 3, according to DSM-IV classification), duration, drug used and dose, response to treatment (0 = no response, 1 = partial, 2 = complete), time of response, concomitant drugs and, for the intake episode, occurrence in the postnatal period. Treatment was coded using the Antidepressant Treatment History Form (ATHF [31]). This is a scale ranging from 1 to 4 where a score of 1 corresponds to an administration of any drug for less than 4 weeks or less than 100 mg of imipramine equivalents, a score of 2 between 100 and 199 mg of imipramine equivalents for 4 weeks or more, a score of 3 between 200 and 299 mg of imipramine equivalents for 4 weeks or more and a score of 4 more than 300 mg imipramine equivalents. We included only those subjects who scored 3 or more at the rating confidence scale (RCS) of the Antidepressant Treatment History Form (range 1–5, from no to high confidence level [31]). Among predictive variables, drug maintenance played a crucial role. A better outcome is observed with regular maintenance. However the retrospective approach hampers a complete assessment of this issue (e.g. stable compliance, blood mood stabilizer levels). Maintenance was performed with mood stabilizers at adequate doses for prophylaxis and antidepressant scoring 2–4 on the ATHF (1–4 for >60 years old patients). We scored this variable as maintenance absent (0) if maintenance was not taken or if it was taken for less than one third of the disease duration, present (2) if taken regularly for more than two thirds of the illness length, partial (1) if taken between one and two thirds and unknown if no or unreliable information (ATHF-RCS <3) was available [6].

Family psychiatric history was collected throughout a structured interview [32] and scored as follows: 0 = no first or second degree relative affected by mood disorders; 1 = one to three second degree affected relatives; 2 = one affected first degree relative or more than three second degree affected relatives; 3 = more than one affected first degree relative. All patients were evaluated on their lifetime symptomatology with the operational criteria (OPCRIT) checklist [33]. This is a 90 item scale which covers most psychotic and affective disorder symptoms. It was originally administered to identify the factor structure of major psychoses [34-36], in the present paper it was used to investigate psychotic features (delusions and/or hallucinations).

### Statistical analyses

Bipolar I and major depressive patients were analyzed separately. Per year rates of excited (manic + mixed) and depressive episodes were compared between PPO and NPPO mood disorders by means of Student's t test. Manic and mixed episodes were pooled together since the retrospective nature of our investigation hampered a clear distinction between these two subtypes. In particular psychotic mania could hardly be distinguished from a delusional mixed state. Confounding variables – the list included age of onset, length of illness, total number of episodes, polarity of the intake episode, family history of psychiatric disorders, psychotic features, comorbid personality disorders and treatment history – asymmetrically distributed between PPO and NPPO samples (p < 0.05) were controlled for using ANCOVA.

The power of our bipolar and MDD samples to detect differences between PPO and NPPO groups was calculated considering an alpha value of 0.01 two tailed. With these parameters in each diagnostic sample we had a high power (0.80) to detect a medium effect size (d = 0.80) that corresponded to a difference of approximately 0.3 points in the episode frequency (14% of variance explained) between the two comparison groups [37]

## Results

### Bipolar sample

In the bipolar sample (N = 123) the mean age at illness onset was 27.6 ± 7.3 years, at study entry it was 42.1 ± 13.5 years. Mean duration of illness was 14.5 ± 11.4 years; family history of mood disturbance were reported in 92.% of probands: 76% of them had one to three cases of affective illness among their second degree relatives, the remaining 16% had more than three cases among their second degree relatives or one affected first degree relative. Mood episodes were on average 2.59 ± 2.07 for excited and 3.37 ± 2.88 for depressive ones.

The characteristics of PPO and NPPO bipolar patients are reported in table I. Women with postpartum onset disorders significantly differed from non-postpartum controls as they were more frequently married (p = 0.002), with a lower recurrence of both excited (p = 0.005) and total affective episodes (p = 0.005). Confounding variables were all symmetrically distributed between comparison groups, so they were not controlled by performing analysis of covariance. One bipolar woman had a single episode disorder. 7 out of 123 bipolar women (5.6%) met criteria for rapid cycling disorder (see above): they were all in the NPPO group.

**Table 1:** Comparison between postpartum and non-postpartum bipolar patients (N = 123)

	**Postpartum (N = 22)**	**Non-postpartum (N = 101)**	**Statistical Analyses**	
Age *(years)*	44.4 ± 12.4	41.7 ± 13.7	t = 0.89	p = 0.372
Marital status *(Single/Married)*^1^	1/21	36/60	X^2 ^= 9.0	p = 0.002*
Education *(years)*	9.5 ± 3.0	10.4 ± 4.3	t = 1.16	p = 0.251
Workers for pay *(Yes/No)*^2^	9/13	56/39	X^2 ^= 2.3	p = 0.125
Personality disorders *(Yes/No)*^3^	9/4	43/28	X^2 ^= 0.3	p = 0.554
Age of onset *(years)*	27.2 ± 6.5	27.6 ± 7.5	t = 0.25	p = 0.800
Polarity at onset *(excited/depressive)*	5/17	35/66	X^2 ^= 1.2	p = 0.279
Length of illness *(years)*	17.2 ± 11.2	13.9 ± 11.5	t = 1.22	p = 0.225
Familiarity *(0 to 3)*	1.00 ± 0.56	1.10 ± 0.85	t = 0.38	p = 0.379
Psychotic symptoms *(Yes/No)*	14/8	82/19	X^2 ^= 3.2	p = 0.071
Maintenance *(0 to 2)*	0.95 ± 0.94	1.33 ± 0.91	t = 1.73	p = 0.086
Any polarity episodes *(total number)*	6.00 ± 3.6	5.96 ± 3.4	t = 0.05	p = 0.961
Any polarity episodes *(number/year)*	0.50 ± 0.34	0.81 ± 0.80	t = 2.85	p = 0.005*
Excited episodes *(number/year)*	0.21 ± 0.26	0.42 ± 0.45	t = 2.89	p = 0.005*
Depressive episodes *(number/year)*	0.29 ± 0.21	0.39 ± 0.51	t = 1.48	p = 0.141

*Significant p < 0.05. ^1^assessed in 118 patients; ^2^assessed in 117 patients; ^3^assessed in 84 patients

### Major depressive sample

Unipolar women (N = 150) mean age at illness onset was 29.4 ± 7.6 years, at study entry it was 48.6 ± 13.1 years. Mean length of illness was 18.7 ± 13.2 years; family history of mood disturbance was reported in 83.% of probands: 66% of them had one to three cases of affective illness among their second degree relatives, the remaining 17% had more than three cases among their second degree relatives or one affected first degree relative. An average of 3.59 ± 3.27 mood disorder episodes were observed in the sample.

The characteristics of PPO and NPPO major depressive patients are reported in table 2. Compared to NPPO controls, PPO patients were more frequently married (p = 0.004) and had a lower prevalence of comorbid personality disorders (p = 0.023). This latter feature was assessed in 96 out of 150 patients (64%). In addition PPO patients showed lower scores on the scale of maintenance treatment (p = 0.002). With respect to illness course, PPO patients had lower recurrence rates than their NPPO counterpart (p = 0.016). This finding was no longer significant after controlling for maintenance (ANCOVA: F = 1.31 p = 0.25).

**Table 2:** Comparison between postpartum and non-postpartum major depressive patients (N = 150)

	**Postpartum (N = 33)**	**Non-postpartum (N = 117)**	**Statistical Analyses**	
Age *(years)*	50.8 ± 13.0	48.0 ± 13.1	t = 0.58	p = 0.565
Marital status *(Single/Married)*^1^	0/33	24/89	X^2 ^= 8.4	p = 0.004*
Education *(years)*	8.6 ± 3.6	9.9 ± 4.2	t = 1.57	p = 0.119
Workers for pay *(Yes/No)*^2^	13/20	57/55	X^2 ^= 1.3	p = 0.245
Personality disorders *(Yes/No)*^3^	7/15	44/30	X^2 ^= 5.2	p = 0.023*
Age of onset *(years)*	30.1 ± 6.6	29.9 ± 7.9	t = 0.14	p = 0.887
Length of illness *(years)*	20.7 ± 11.9	18.1 ± 13.5	t = 1.02	p = 0.308
Familiarity *(0 to 3)*	1.03 ± 0.5	1.00 ± 0.6	t = 0.27	p = 0.786
Psychotic symptoms *(Yes/No)*	11/22	34/83	X^2 ^= 0.3	p = 0.562
Maintenance *(0 to 2)*	0.36 ± 0.7	0.85 ± 0.9	t = 3.15	p = 0.002*
Depressive episodes *(total number)*	3.06 ± 2.4	3.74 ± 3.5	t = 1.06	p = 0.291
Depressive episodes *(number/year)*	0.26 ± 0.3	0.41 ± 0.4	t = 2.45	p = 0.016*

*Significant p < 0.05. ^1^assessed in 146 patients; ^2^assessed in 145 patients; ^3^assessed in 96 patients

27 major depressive women were diagnosed with single episode disorder, 7 in the PPO (21%) and 20 in the NPPO (17%) group (p = 0.59) Their mean length of illness was 12.7 ± 13.7 years compared to 17.3 ± 12.4 years of subjects with recurrent episodes (p = 0.07). No major depressive woman met criteria for rapid cycling disorder.

## Discussion

This study addressed the topic of predicting the course of mood disorders at early stages. For researchers, specific time course predictors might help in identifying endophenotypes for biological and genetic investigations [38]. For clinicians, the early recognition of disorders with a relatively good prognosis would warrant less intensive treatment and, as such, a minor burden of side effects.

Among possible early course predictors we focused on postpartum onset because:

1) pregnancy and the postpartum period are distinct neurobiological states with regard to brain function and the incidence of psychiatric disorders is different during these periods [39,40];

2) to know how the disease may evolve is even more important for a mother who has to take care of her child in the early "critical" years of life.

Therefore we retrospectively evaluated illness course in mood disorders with and without postpartum onset. Since bipolar disorders have repeatedly been associated with a poorer prognosis [3,41], bipolar and major depressed patients were analyzed separately.

### Bipolar sample

The most striking result was the lower recurrence of total affective episodes and excited (manic/mixed) episodes in PPO bipolar I disorder. In addition all bipolar women with a rapid cycling disorder (7 out of 133) did not have their intake episode during the postpartum period. Altogether these findings suggest that in bipolar I disorder postpartum onset may predict a time course characterized by a lower recurrence. Whether this implies a better outcome in terms of social functioning and quality of life was an issue not addressed in the present study. We only found that PPO bipolars were more frequently married compared to NPPO comparison patients, while the two groups were similar in education level and employment status. Future research in this field should incorporate the assessment of social adjustment and quality of life. On the contrary, excited depressive episodes had a similar recurrence rate in PPO and NPPO bipolar disorders. A possible explanation for this fact is based on recent evidence that postpartum relapse in bipolar women is usually depressive [42]. Another possible explanation is that in our PPO group some bipolars might have an overall higher threshold for relapse with the majority of episodes occurring only in the postpartum period: such individuals are expected to have fewer episodes during illness course, almost exclusively depressive and in the postpartum period. This would lead to a slight decrease in the rates of depressive episodes but a significant decrease in manic episodes. Such a hypothesis could not be verified here as we had no information on postpartum episodes other than the first one. Another explanation is that our findings could be artefacts due to an asymmetric distribution of established course predictors. Of such variables, anxiety disorders [43,44], alcohol/substance abuse [45-47] and inter-episode residual symptoms [48-50] were exclusion criteria. Other confounding factors such as personality disorders [15,51] and clinical features including age [52] and polarity at onset [4,5,53], psychotic manifestations [10,11], length of illness and number of episodes prior to study entry [2,3,54] were similarly distributed between PPO and NPPO samples. Drug maintenance was a crucial issue in this study, although its effect is not clear. Lithium prophylaxis in bipolar patients produces a significant reduction of the mean number of episodes [55,56]. Unfortunately, even with sustained lithium prophylaxis, the likelihood of at least one recurrence exceeded 70% within 5 years of recovery [57]. Moreover some have suggested that antidepressant drugs may increase the recurrence rate in bipolars [58,59]. In our bipolar sample drug maintenance could not bias results as it did not significantly differ between PPO and NPPO subjects. However there was no available information on patients' compliance (e.g plasma levels of antidepressants [60] and mood stabilizers [61]).

### Depressive sample

Patients with PPO major depressive disorder experienced fewer episodes during illness course compared to the NPPO sample. The latter subjects were more likely to be on maintenance treatment which could have lowered their "natural" relapse rate. This strengthen the hypothesis that PPO depressive disorder has a better course. However the control for maintenance treatment greatly reduced the difference between PPO and NPPO patients. It is a paradoxical finding whose most likely implication is the lack of a protective effect of maintenance therapy in our MDD sample. It should be noted that PPO unipolar women were also associated with a lower prevalence of personality disorders. This may (at least partially) explain why they were less likely to relapse given the known negative impact of axis II comorbidity on the outcome of mood disorders [51]. The retrospective design of the study could bias data collection toward a decreased detection of past episodes and unreliable estimates of clinical variables. In particular we found a very low prevalence of rapid cycling. To control this bias we used a set of strategies: clinical information was obtained by interviewing patients, their relatives and previous health professionals and, whenever possible, by examining records [29]; a blind experienced psychiatrist reviewed charts; unreliability was assessed and considered as exclusion criterion. Another caveat is related to the atypical features of our sample collected in a tertiary care setting. These are illustrated by the high percentage of bipolar disorders, the high rate of positive family history and the fact that this value was not different between BPD and MDD patients. Such flaws limit the generalizability of our results and emphasize the need for further prospective studies.

## Conclusion

Our preliminary data suggest that postpartum onset may be associated with a less severe time course of mood disorders. The present study design does not allow to suggest postpartum affective disorder as a stand alone diagnostic category. In fact an onset of the disease during the postpartum period is not a good criterion to exclude "pseudopostpartum" patients, that is bipolar and major depressive patients with episodes fortuitously occurring during postpartum. Furthermore not all women in our control group were parous, a prerequisite to understand their susceptibility to postpartum recurrence.

## Competing interests

The author(s) declare that they have no competing interests.

## Authors' contributions

AS coordinated the study, and participated in its design. PO performed the statistical analysis and drafted the manuscript. CC coordinated patient recruitment and assessment. All authors read and approved the final manuscript.

## Pre-publication history

The pre-publication history for this paper can be accessed here:


